# The Influence of the CHIEF Pathway on Colorectal Cancer-Specific Mortality

**DOI:** 10.1371/journal.pone.0116169

**Published:** 2014-12-26

**Authors:** Martha L. Slattery, Abbie Lundgreen

**Affiliations:** Department of Internal Medicine, University of Utah Health Sciences Center, 383 Colorow Building, Salt Lake City, Utah, United States of America; Centro Nacional de Investigaciones Oncológicas (CNIO), Spain

## Abstract

Many components of the CHIEF (Convergence of Hormones, Inflammation, and Energy Related Factors) pathway could influence survival given their involvement in cell growth, apoptosis, angiogenesis, and tumor invasion stimulation. We used ARTP (Adaptive Rank Truncation Product) to test if genes in the pathway were associated with colorectal cancer-specific mortality. Colon cancer (n = 1555) and rectal cancer (n = 754) cases were followed over five years. Age, center, stage at diagnosis, and tumor molecular phenotype were considered when calculating ARTP p values. A polygenic risk score was used to summarize the magnitude of risk associated with this pathway. The JAK/STAT/SOC was significant for colon cancer survival (P_ARTP_ = 0.035). Fifteen genes (*DUSP2, INFGR1, IL6, IRF2, JAK2, MAP3K10, MMP1, NFkB1A, NOS2A, PIK3CA, SEPX1, SMAD3, TLR2, TYK2*, and *VDR*) were associated with colon cancer mortality (P_ARTP_ <0.05); *JAK2* (P_ARTP_  = 0.0086), *PIK3CA* (P_ARTP_ = 0.0098), and *SMAD3* (P_ARTP_ = 0.0059) had the strongest associations. Over 40 SNPs were significantly associated with survival within the 15 significant genes (P_ARTP_<0.05). *SMAD3* had the strongest association with survival (HR_GG_ 2.46 95% CI 1.44,4.21 P_Ttrnd_ = 0.0002). Seven genes (*IL2RA, IL8RA, IL8RB, IRF2, RAF1, RUNX3*, and *SEPX1*) were significantly associated with rectal cancer (P_ARTP_<0.05). The HR for colorectal cancer-specific mortality among colon cancer cases in the upper at-risk alleles group was 11.81 (95% CI 7.07, 19. 74) and was 10.99 (95% CI 5.30, 22.78) for rectal cancer. These results suggest that several genes in the CHIEF pathway are important for colorectal cancer survival; the risk associated with the pathway merits validation in other studies.

## Introduction

The CHIEF (Convergence of Hormones, Inflammation, and Energy Related Factors) pathway integrates elements central to the etiology of colorectal cancer (CRC) [1]. The pathway was developed based on our knowledge of the epidemiology of CRC and genes that may influence cancer risk through major components of the pathway, including hormones, inflammation, and energy-related factors [1]. Many genes in the pathway could influence tumor progression and prognosis given their involvement cell growth, apoptosis, promotion of inflammation and angiogenesis, immune response, and stimulation of tumor invasion and metastasis [2]. The main trunk of the pathway contains a serine/threonine protein kinase 11 (STK11 or LKB1), mammalian target of rapamycin (MTOR), and the tumor suppressor PTEN (phosphatase tensin homolog deleted on chromosome 10). STK11 responds to changes in cellular energy balance (ATP levels) [3], [4] and governs whole body insulin sensitivity [5], [6]. NFκB is an important nuclear transcription factor that regulates cytokines and is critical for the regulation of tumorigenesis, cell proliferation, apoptosis, response to oxidative stress, and inflammation while vascular endothelial growth factor (VEGF) plays an important role in regulation of cell growth signaling and is a major mediator of tumor angiogenesis [7] [8].

Cytokines such as interleukins, TGFβ-signaling pathway, interferons, and tumor necrosis factor (TNF), are key elements of the inflammatory process in the CHIEF pathway. The TGF-β-signaling pathway is involved in all aspects of tumorigenesis, including stimulation of tumor invasion and metastasis [2]. Signal transduction and activation of transcription (STAT) and mitogen-activated kinases (MAPK) genes are involved in both inflammation and metabolic signaling associated with hormones and energy-related factors. MAPKs serve as an integration point for multiple biological signals and are involved in a variety of cellular processes such as proliferation. Angiogenesis and inflammation are hallmark features of tumorigenesis [9] as well as key elements in the CHIEF pathway, thus it is reasonable to hypothesis that pathway influences survival.

In this paper, we summarize the significance of this pathway as it relates to survival after being diagnosed with colon or rectal cancer using Adaptive Rank Truncation Product (ARTP), building on our previous work that evaluated the pathway with colon and rectal cancer risk where we documented overall risk as well as risk specific to tumor molecular phenotype [10]. This statistical program utilizes a permutation method that allows us to summarize across genes within sub-pathways of the overall pathway to estimate the association with survival of the pathway, genes, and SNPs within the pathway. To further estimate the magnitude of the association of this pathway on survival, we utilize a polygenic risk score that is based on the permutated ARTP findings.

## Methods

Two study populations are included in these analyses. The first study, a population-based case-control study of colon cancer, included cases (n = 1,555 with complete genotype data) identified between October 1, 1991 and September 30, 1994 living in the Twin Cities Metropolitan Area or a seven-county area of Utah or enrolled in the Kaiser Permanente Medical Care Program of Northern California (KPMCP) [11]. The second study, with identical data collection methods, included cases with cancer of the rectosigmoid junction or rectum (n = 754 cases with complete genotype data) who were identified between May 1997 and May 2001 in Utah and at the KPMCP [12]. Eligible cases were between 30 and 79 years of age at the time of diagnosis, living in the study geographic area, English speaking, mentally competent to complete the interview, and with no previous history of CRC, and no previous diagnosis of familial adenomatous polyposis, ulcerative colitis, or Crohn's disease. Cases who did not meet these criteria were ineligible as were individuals who were not black, white, Hispanic, or Asian (for the rectal cancer study). All study participants provided written informed consent on Institutional Review Board approved consent forms prior to completing the study questionnaire; the consent form and study protocol was approved by the Institutional Review Board on Human Subjects at the University of Utah, Kaiser Permanente Medical Research Program, and the University of Minnesota.

### Tumor Registry Data

Tumor registry data were obtained to determine disease stage at diagnosis and months of survival after diagnosis. Disease stage was categorized using the sixth edition of the American Joint Committee on Cancer (AJCC) staging criteria. One pathologist in Utah did all disease staging. Local tumor registries provided information on patient follow-up including vital status, cause of death, and contributing cause of death. Follow-up was obtained for all study participants and was terminated for the Colon Cancer Study in 2000 and for the Rectal Cancer Study in 2007. At that time all study participants had over five years of follow-up.

### Tumor Marker Data

Tumors were defined by specific molecular alterations: any *TP53* mutation; any *KRAS* mutation; MSI+; and CpG Island Methylator Phenotype (CIMP). CIMP status was based on the classic panel and defined as positive if at least two of five markers were methylated [13]. Microsatellite instability (MSI) was based on *BAT26*, *TGFβRII*, and a panel of 10 tetranucleotide repeats that has been shown to correlate highly with the Bethesda Panel [14]; our study was done prior to the Bethesda Panel development. These data are included in analysis since we have shown that tumor molecular phenotype influences survival and is associated with SNPs in this pathway [10], [15]

### TagSNP Selection and Genotyping

TagSNPs were selected using the following parameters: r^2^ = 0.8 defined LD blocks using a Caucasian LD map, minor allele frequency (MAF)>0.1, range = −1500 bps from the initiation codon to +1500 bps from the termination codon, and 1 SNP/LD bin. All markers were genotyped using a custom multiplexed bead array assay format based on GoldenGate chemistry (Illumina, San Diego, California). A genotyping call rate of 99.85% was attained. Blinded internal replicates represented 4.4% of the sample set. The duplicate concordance rate was 100.00%. S1 Table list all genes included in the sub-pathway while S2 Table list number of SNPs assessed for each gene and the P_ARTP_ value for each gene on the platform. We analyzed data from 155 genes which included 10 genes that were previously assessed in our lab (*VDR*, *ESR1*, *ESR2*, *AR*, *IGF1*, *IGF1R*, *IGFBR3*, *IRS1*, *IRS2*, and *PPARG*) along with 145 genes from the Illumina platform. The initial platform included 1536 SNPs, of these, 1381 were successfully analyzed by Illumina. We included in our analysis only those SNPs were>95% of the population had results, leaving 1246 SNPs for analysis No imputation was done.

### Statistical Methods

The goal of the analysis was to evaluate the overall associations between genes and pathways as they relate to colon and rectal cancer survival. To do this, we used ARTP, a statistical program that utilizes a highly efficient permutation algorithm to determine significance at the gene, sub-pathway, and pathway level for survival after diagnosis with colon or rectal cancer [16]. Vital status and survival months were permuted 10,000 times within R version 3.0.2 (R Foundation for Statistical Computing, Vienna, Austria). Since our focus was on colorectal cancer-specific mortality, people who died from other causes or who were lost to follow-up were censored at the date of death or last contact. Months of survival were calculated from date of diagnosis until end of follow-up or date of last contact. Cox Proportional Hazards models were adjusted for age, race/ethnicity, sex, AJCC stage, and tumor molecular phenotype. Tumors were defined by specific molecular alterations: any *TP53* mutation; any *KRAS* mutation; MSI+; and CIMP high. As the proportion of MSI+ tumors in the rectal cases was <3% [17], we did not include these tumor markers as an adjustment variable for rectal cancer. Associations with SNPs within ARTP were assessed assuming an additive model unless a preliminary check of the hazard ratios indicated a dominant or recessive mode of inheritance. For SNPs with gene p values <0.05 that were associated with colon or rectal cancer based on ARTP results, we report Hazard Ratios (HR) and 95% confidence intervals (CIs) assessed from Cox Proportional Hazard models in SAS to show the magnitude of the association between these SNPs and hazard of dying after diagnosis with colon or rectal cancer; we also report p values for likelihood ratio test (LRT). We include those genes which contributed to the ARTP permutated gene p value for reference since they could possibly indicate greater significance and are of interest for replication elsewhere. We did not further adjust SNP associations for multiple comparisons since our analytic approach is top down: looking at the overall pathway (where number of genes are adjusted), genes (where number of SNPs are adjusted), and SNPs that contribute to significant permutated P_ARTP_ values. Genes were assigned to only one sub-pathway prior to the hierarchical analyses. However, we realize many genes could function in other sub-pathways to which they were not assigned for analysis.

To summarize the risk associated with the CHIEF pathway, we calculated polygenic summary scores. To conservatively estimate risk, we included in the risk models SNPs from genes where the gene ARTP p values were 0.10 or less and the SNP p values within those genes were 0.10 or less. Our analysis includes SNPs with p<0.10 only from those genes where the P_ARTP_ was <0.10. Thus, we include SNPs that were not statistically significant and we omit SNPs that were statistically significant in genes where the P_ARTP_ was>0.10. Since genes are associated with multiple sub-pathways, we did not restrict to genes where the sub-pathway was significant. If SNPs within the same gene had r^2^ values of 0.80 or greater only one SNP was included in the model. Risk was modeled using at-risk alleles, using all genotypes with the low-risk genotype or referent group as zero. For the co-dominant or additive model a score of zero, one, or two was assigned relative to the number of at-risk alleles, while scores of zero or two were assigned for the dominant and recessive models in order to capture the risk associated with the various genotypes. Polygenic scores were then used to summarize risk across the genes and SNPs to better capture the risk associated with the pathway.

## Results

The majority of study participants were over 60 years of age, were non-Hispanic white, and male (Table 1). Most cases were diagnosed with an AJCC Stage 1 or 2 tumor. At the end of follow-up roughly 35% of study participants had died. The overall pathway was not statistically significantly associated with survival for either colon or rectal cancer (Table 2). However, the JAK/STAT/SOC was significant for colon cancer survival (P_ARTP_ = 0.035) and the interleukin pathway was of borderline significance for rectal cancer (P_ARTP_ = 0.06).

**Table 1:** Description of study population.

		Colon	Rectal
		n (%)	n (%)
Age	30–39	23 (1.48)	19 (2.52)
	40–49	102 (6.57)	96 (12.73)
	50–59	289 (18.61)	196 (25.99)
	60–69	537 (34.58)	250 (33.16)
	70–79	602 (38.76)	193 (25.60)
Center	Utah	249 (16.03)	274 (36.34)
	KPMCP	742 (47.78)	480 (63.66)
	Minnesota	562 (36.19)	
Race/Ethnicity	NHW	1426 (91.82)	625 (82.89)
	Hispanic	59 (3.80)	61 (8.09)
	Black	68 (4.38)	29 (3.85)
	Asian		39 (5.17)
Sex	Male	868 (55.89)	451 (59.81)
	Female	685 (44.11)	303 (40.19)
Tumor Molecular Phenotypes	CIMP+	272 (26.96)	59 (11.11)
	*KRAS2* Mutation	348 (31.93)	173 (29.37)
	*TP53* Mutation	515 (45.90)	277 (49.64)
	MSI Unstable	185 (15.76)	14 (2.39)
AJCC Stage	1	468 (30.14)	381 (50.53)
	2	404 (26.01)	124 (16.45)
	3	374 (24.08)	175 (23.21)
	4	128 (8.24)	57 (7.56)
	Unknown	179 (11.53)	17 (2.25)
Vital Status	Dead	520 (33.48)	259 (34.35)
	Alive^1^	1033 (66.52)	495 (65.65)
Cause of Death	Colorectal Cancer	309 (59.42)	171 (66.02)
	Other Cancer	58 (11.15)	14 (5.41)
	Non-Cancer	90 (17.31)	37 (14.29)
	Unspecified/Unknown	63 (12.12)	37 (14.29)
Percent Five-Year Survival^2^		65.71%	73.09%
Median Survival Time (months)^3^		62	74

1 Includes cases lost to follow-up within five years of diagnosis.

2 Excludes cases lost to follow-up within five years of diagnosis.

3 Time from diagnosis to death or last follow-up.

**Table 2:** Overall pathway P_ARTP_ 1.

	Colon		Rectal	
Sub-Pathway	Sub-Pathway	Pathway	Sub-Pathway	Pathway
	P_ARTP_	P_ARTP_	P_ARTP_	P_ARTP_
Angiogenesis	0.2426	0.2479	0.8865	0.6248
Hormone/Insulin/Growth	0.4030		0.7416	
Interferons	0.0770		0.1720	
Interleukins	0.4662		0.0609	
Jak/Stat/Socs	**0.0353**		0.5152	
Pathway Core	0.2036		0.7114	
MAP Kinase (MAPK)	0.3160		0.3529	
Selenoproteins	0.1834		0.3659	
Telomere	0.5166		0.9729	
TGFβ	0.1503		0.4647	
Toll-Like Receptors (TLR)	0.1109		0.9874	
Tumor Necrosis Factor (TNF)	0.8566		0.1712	

1 Adjusted for age, study center, race/ethnicity, sex, AJCC stage, and tumor markers: CIMP, *KRAS*, *TP53*; MSI for colon only. ARTP p values based on 10,000 permutations.

However several genes within the sub-pathways were significant for colon (Table 3) and rectal (Table 4) cancer mortality. Fifteen genes (*DUSP2, INFGR1, IL6, IRF2, JAK2, MAP3K10, MMP1, NFkB1A, NOS2A, PIK3CA, SEPX1, SMAD3, TLR2, TYK2*, and *VDR*) were significantly associated with colon cancer mortality at the <0.05 level; an additional 15 genes had gene P_ARTP_ values between 0.05 and 0.10 (see S3 Table). The genes that were most significantly associated with survival were *JAK2* (P_ARTP_ = 0.0086), *PIK3CA* (P_ARTP_ = 0.0098), and *SMAD3* (P_ARTP_ = 0.0059). Over 40 SNPs were significantly associated with survival within the 15 significant genes (P_ARTP_<0.05). Of these SNPs, *SMAD3* had the strongest association with survival (HR_GG_ 2.46 95% CI 1.44,4.21 P_LRT_ = 0.0002). Ten SNPs in five genes had P values less than 0.005, including *IL6* rs1800796 (HR_GG_ 0.55 95% CI 0.36, 0.84), *IRF2* rs12504466 (HR_TT_ 1.51 95% CI 1.14,1.99), rs793814 (HR_TT/AA_ 0.57 95% CI 0.39,0.83), and rs3775582 (HR_AA/AT_ 0.67 95% CI 0.50,0.89), *JAK2* rs7043371 (HR_AT/TT_ 0.63 95% CI 0.47,0.84) and rs10815160 (HR_TT_ 1.62 95% CI 1.07,2.47), *SEPX1* rs732510 (HR_AA/AG_ 1.47 95% CI 1.13,1.90), and *SMAD3* rs893473 (HR_CC_ 1.45 95% CI 1.14,1.83) rs1866317 (HR_CC_ 1.47 95% CI 1.14,1.90), and rs12708492 (HR_CC_ 1.52 95% CI 1.16,2.00).

**Table 3:** Genes and related SNPs associated with colorectal cancer-specific mortality among patients diagnosed with colon cancer (gene P_ARTP_≤0.05; SNP P_trend_≤0.10).

Gene	P_ARTP_	SNP	Genotype	HR (95%CI)^1^	P_trend_
*DUSP2*	**0.0225**	rs1724120	AA vs. GG/GA	0.72 (0.54, 0.96)	0.0199
*IFNGR1*	**0.0121**	rs3799488	TC/CC vs. TT	1.30 (0.98, 1.72)	0.0772
		rs9376267	CT/TT vs. CC	1.37 (1.09, 1.73)	0.0079
		rs1327474	GG vs. AA/AG	0.69 (0.50, 0.94)	0.0158
*IL6*	**0.0417**	rs1800796	GC/CC vs. GG	0.55 (0.36, 0.84)	0.0032
*IRF2*	**0.0207**	rs6856910	CC vs. TT	1.42 (0.99, 2.04)	0.0835
		rs793777	GG vs. CC	0.67 (0.46, 0.98)	0.0426
		rs2797507	CA/AA vs. CC	0.77 (0.61, 0.98)	0.0380
		rs12504466	TC/CC vs. TT	1.51 (1.14, 1.99)	0.0027
		rs793814	AA vs. TT/TA	0.57 (0.39, 0.83)	0.0018
		rs7655800	AG/GG vs. AA	1.33 (1.04, 1.70)	0.0234
		rs9684244	CC vs. GG	0.56 (0.37, 0.84)	0.0124
		rs13139310	AA vs. GG	0.35 (0.16, 0.74)	0.0220
		rs11723606	TT vs. CC	0.45 (0.24, 0.86)	0.0341
		rs13116389	GT/TT vs. GG	1.38 (1.09, 1.75)	0.0073
		rs793801	AA vs. GG/GA	1.39 (1.01, 1.91)	0.0506
		rs3775582	GA/AA vs. GG	0.67 (0.50, 0.89)	0.0038
		rs1044873	CT/TT vs. CC	1.32 (1.04, 1.68)	0.0231
*JAK2*	**0.0086**	rs1887429	GT/TT vs. GG	1.34 (1.07, 1.69)	0.0113
		rs7043371	TT vs. AA/AT	0.63 (0.47, 0.84)	0.0010
		rs10974947	AA vs. GG	1.34 (0.86, 2.10)	0.0319
		rs3780379	GA/AA vs. GG	1.32 (1.04, 1.67)	0.0221
		rs10815160	GG vs. TT	1.62 (1.07, 2.47)	0.0017
*MAP3K10*	**0.0306**	rs1129156	TT vs. CC	1.49 (0.89, 2.52)	0.0073
*MMP1*	**0.0289**	rs470215	CC vs. TT	1.45 (0.99, 2.12)	0.0278
*NFKBIA*	**0.0252**	rs696	AA vs. GG	1.41 (1.00, 1.99)	0.0696
		rs2233409	TT vs. CC	0.62 (0.37, 1.03)	0.0562
		rs3138053	GG vs. AA	0.56 (0.35, 0.90)	0.0177
*NOS2A*	**0.0421**	rs7406657	CC vs. GG	0.59 (0.32, 1.09)	0.0061
		rs9906835	GG vs. AA	0.62 (0.43, 0.89)	0.0105
		rs2297516	CC vs. AA	0.59 (0.40, 0.86)	0.0095
*PIK3CA*	**0.0098**	rs2699905	GA/AA vs. GG	0.73 (0.58, 0.93)	0.0101
		rs7640662	CG/GG vs. CC	0.71 (0.54, 0.94)	0.0154
		rs2677760	CC vs. TT/TC	1.43 (1.11, 1.83)	0.0067
		rs1607237	CC vs. TT/TC	1.45 (1.10, 1.92)	0.0104
*SEPX1*	**0.0217**	rs732510	GG vs. AA/AG	1.47 (1.13, 1.90)	0.0049
*SMAD3*	**0.0059**	rs1498506	CC vs. AA	0.69 (0.48, 0.99)	0.0837
		rs9972423	AA vs. TT	0.82 (0.56, 1.19)	0.0950
		rs2118611	GG vs. AA	1.89 (1.19, 2.99)	0.0188
		rs11071933	GG vs. CC	1.60 (1.15, 2.24)	0.0111
		rs7163381	AA vs. GG	1.67 (1.09, 2.58)	0.0113
		rs4776892	TT vs. AA	1.64 (0.93, 2.91)	0.0292
		rs2414937	CC vs. GG	2.46 (1.44, 4.21)	0.0002
		rs745103	CC vs. TT	1.50 (1.08, 2.08)	0.0186
		rs893473	CT/TT vs. CC	1.45 (1.14, 1.83)	0.0024
		rs1866317	CG/GG vs. CC	1.47 (1.14, 1.90)	0.0040
		rs4601989	TT vs. CC	0.48 (0.24, 0.93)	0.0719
		rs11639295	TT vs. CC/CT	0.54 (0.33, 0.89)	0.0083
		rs12708492	CT/TT vs. CC	1.52 (1.16, 2.00)	0.0019
*TLR2*	**0.0302**	rs5743704	CA/AA vs. CC	1.80 (1.20, 2.68)	0.0077
		rs5743708	GA/AA vs. GG	1.77 (1.15, 2.72)	0.0160
*TYK2*	**0.0178**	rs12720356	TG/GG vs. TT	1.30 (0.96, 1.76)	0.0933
		rs280521	GA/AA vs. GG	0.69 (0.52, 0.92)	0.0078
		rs280523	GA/AA vs. GG	0.59 (0.38, 0.91)	0.0105
*VDR*	**0.0499**	VDR_Bsm1	BB vs. bb	1.50 (1.06, 2.12)	0.0453
		VDR_Fok1	ff vs. FF	1.47 (1.01, 2.15)	0.0709
		VDR_Poly	SS vs. LL	1.47 (1.03, 2.10)	0.0483

1 Hazard Ratio (HR) and 95% Confidence Intervals (CI) adjusted for age, study center, race/ethnicity, sex, AJCC stage, and tumor molecular phenotype: MSI, CIMP, *KRAS*, and *TP53*. P_ARTP_ based on 10,000 permutations.

**Table 4:** Genes and related SNPs associated with colorectal cancer-specific mortality among patients diagnosed with rectal cancer (gene P_ARTP_≤0.05; SNP P_trend_≤0.10).

Gene	P_ARTP_	SNP	Genotype	HR (95%CI)^1^	P_trend_
*IL2RA*	**0.0216**	rs2386841	AA vs. CC	3.10 (1.50, 6.41)	0.0298
		rs7072398	GA/AA vs. GG	0.62 (0.45, 0.85)	0.0035
		rs11256456	CC vs. TT	1.90 (0.97, 3.70)	0.0049
		rs11256457	CG/GG vs. CC	0.70 (0.51, 0.96)	0.0282
		rs6602398	GT/TT vs. GG	0.76 (0.56, 1.04)	0.0861
		rs11256497	AA vs. GG	0.59 (0.34, 1.01)	0.0588
		rs791587	AA vs. GG	0.57 (0.36, 0.90)	0.0129
		rs10905669	TT vs. CC	1.73 (0.93, 3.21)	0.0054
		rs2476491	AA vs. TT	0.56 (0.29, 1.09)	0.0210
		rs2256774	AG/GG vs. AA	0.68 (0.50, 0.93)	0.0153
		rs706779	GG vs. AA	0.64 (0.41, 1.01)	0.0388
		rs706778	GA/AA vs. GG	1.58 (1.10, 2.26)	0.0103
		rs3118470	TC/CC vs. TT	1.45 (1.06, 2.00)	0.0201
*IL8RA*	**0.0189**	rs1008563	CT/TT vs. CC	0.71 (0.52, 0.98)	0.0368
		rs1008562	GG vs. CC	1.60 (1.04, 2.46)	0.0278
		rs16858808	CT/TT vs. CC	0.51 (0.23, 1.11)	0.0637
		rs16858811	TG/GG vs. TT	0.52 (0.25, 1.08)	0.0571
*IL8RB*	**0.0306**	rs4674258	CT/TT vs. CC	0.72 (0.52, 0.99)	0.0436
		rs1126579	TT vs. CC	1.60 (1.05, 2.46)	0.0235
*IRF2*	**0.0091**	rs809909	TA/AA vs. TT	0.76 (0.56, 1.05)	0.0986
		rs10009261	TT vs. CC	1.52 (0.93, 2.49)	0.0730
		rs1425551	CC vs. AA/AC	1.51 (1.03, 2.20)	0.0396
		rs807684	GG vs. AA/AG	0.31 (0.14, 0.67)	0.0005
		rs3756094	AA vs. GG/GA	0.37 (0.20, 0.67)	0.0003
*RAF1*	**0.0158**	rs3729931	TT vs. CC	0.65 (0.39, 1.09)	0.0690
		rs9809501	TG/GG vs. TT	0.62 (0.40, 0.95)	0.0229
		rs11923427	CG/GG vs. CC	0.58 (0.40, 0.85)	0.0039
		rs11711419	AT/TT vs. AA	0.71 (0.50, 1.00)	0.0452
		rs4684871	GG vs. AA	0.56 (0.33, 0.96)	0.0260
		rs904453	AA vs. CC	1.73 (1.12, 2.68)	0.0132
*RUNX3*	**0.0244**	rs7517302	CC vs. TT	1.77 (1.15, 2.71)	0.0098
		rs2135756	GG vs. AA/AG	0.54 (0.35, 0.82)	0.0022
*SEPX1*	**0.0311**	rs13331553	TC/CC vs. TT	1.45 (1.06, 1.98)	0.0202
		rs732510	GG vs. AA/AG	1.47 (1.04, 2.07)	0.0335

1 Hazard Ratio (HR) and 95% Confidence Intervals (CI) adjusted for age, study center, race/ethnicity, sex, AJCC stage, and tumor molecular phenotype: CIMP, *KRAS*, and *TP53*. P_ARTP_ based on 10,000 permutations.

Fewer genes were associated with survival after diagnosis with rectal cancer than for colon cancer (Table 4). Seven genes (*IL2RA*, *IL8RA*, *IL8RB*, *IRF2*, *RAF1*, *RUNX3*, and *SEPX1*) had P_ARTP_ values <0.05, while nine genes (*BMP1*, *BMPR1A*, *ESR2*, *IL1A*, *IL3*, *PRKAG2*, *SOCS1*, *STK11*, and *TSC2*) had P_ARTP_ values between 0.05 and 0.10 (S4 Table). *SEPX1* rs732510 was associated with both colon and rectal mortality with similar magnitudes of association. Several SNPs in the genes with P_ARTP_<0.05 also had linear trend P values of <0.005, including *IR2RA* rs7072398 (HR_GG_ 0.62 95% CI 0.45,0.86), *IRF2* rs807684 (HR_AA/AG_ 0.31 95% CI 0.14, 0.67 P_LRT_ 0.0005) and rs3756094 (HR_GG/GA_ 0.37 95% CI 0.20,0.67 P_LRT_ 0.0003), *RAF1* rs11923427 (HR_CC_ 0.58 95% CI 0.40,0.65), and *RUNX3* rs2135756 (HR_AA/AG_ 0.54 95% CI 0.35,0.82).

**Figure 1:**
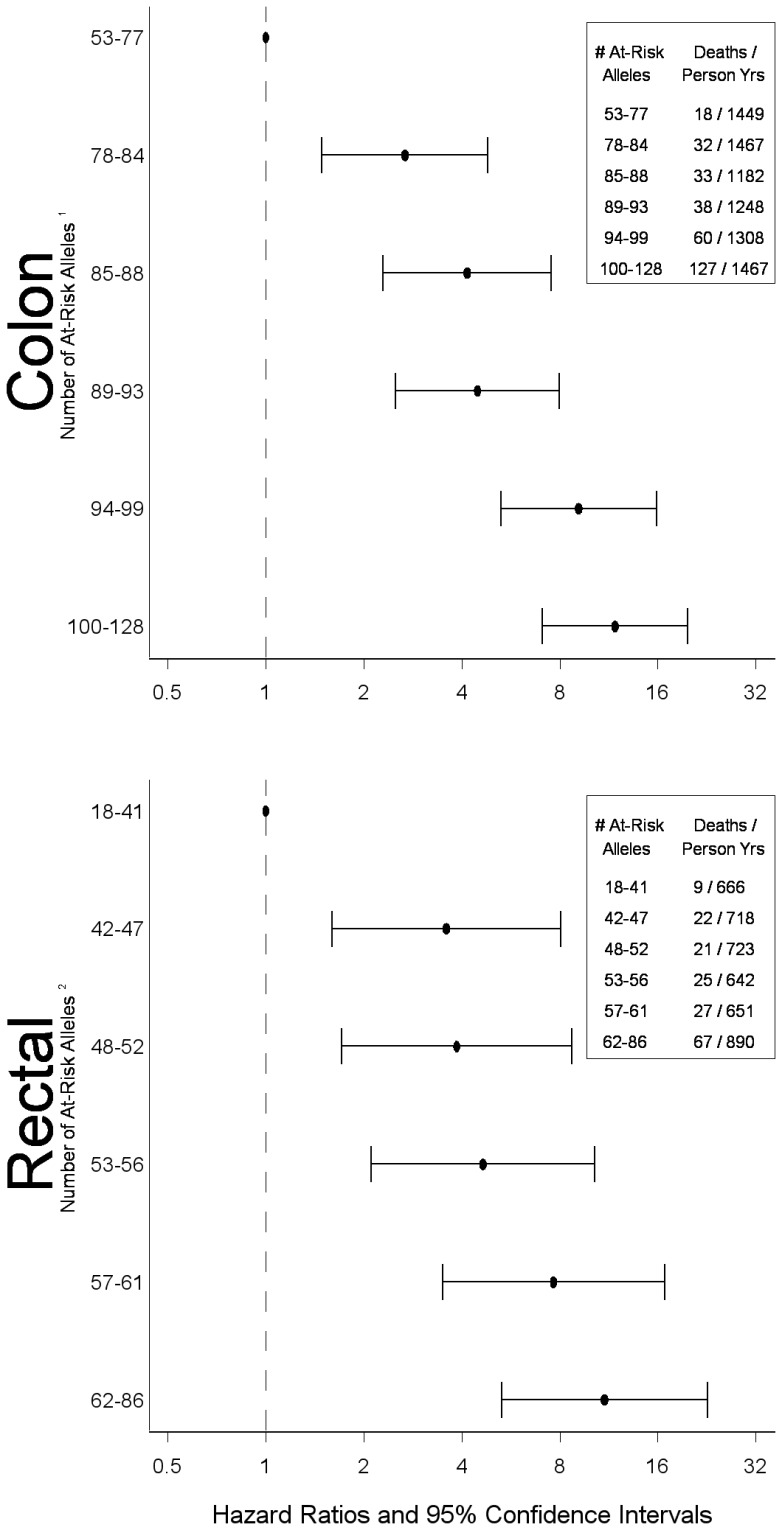
Polygenic summary score associated with CHIEF pathway for colorectal cancer survival. ^1^SNPs included in score: *BMP2* rs1979855, rs3178250, *BMPR1A* rs7895217, rs10887668, *BMPR1B* rs10049681, rs4699673, rs12508087, rs9307147, rs4490463, rs2120834, *DUSP2* rs1724120, EIF4EBP3 rs250425, *IFNGR1* rs3799488, rs9376267, rs1327474, *IGF1*, *IKBKB* rs5029748, rs10958713, *IL1B* rs1143627, rs1143623, *IL6* rs1800796, *IRF2* rs6856910, rs793777, rs2797507, rs12504466, rs793814, rs7655800, rs9684244, rs13139310, rs13116389, rs793801, rs3775582, *IRF8* rs305083, rs305080, rs11649318, rs13338943, rs10514611, rs1044873, *JAK2* rs1887429, rs7043371, rs10974947, rs3780379, rs10815160, *JUNB* rs2229510, *MAP3K10* rs1129156, *MMP1* rs470215, *MMP3* rs3025066, *NFKBIA* rs696, rs2233409, rs3138053, *NOS2A* rs7406657, rs2297516, *PIK3CA* rs2699905, rs7640662, rs2677760, rs1607237, *RPS6KA2* rs2049956, rs1894660, rs6918886, rs932356, rs9459715, rs1883361, rs4710090, rs661325, rs2345067, rs2072638, rs1309150, rs7745781, *SEP15* rs9433110, *SEPX1* rs732510, *SMAD3* rs1498506, rs9972423, rs2118611, rs11071933, rs7163381, rs4776892, rs2414937, rs745103, rs893473, rs1866317, rs4601989, rs11639295, rs12708492, *SOCS1* rs4780355, *STAT3* rs1053005, rs2293152, rs8069645, *STAT5A* rs12601982, *TLR2* rs5743704, rs5743708, *TYK2* rs12720356, rs280521, rs280523, *VDR*_Fok1, *VDR*_Poly. 2SNPs included in score: *BMP1* rs12114940, rs3924229, rs3857979, *BMPR1A* rs7088641, rs2168730, rs7895217, rs4934275, *ESR2*_Rsa, *IL1A* rs3783546, *IL2RA* rs2386841, rs7072398, rs11256456, rs11256457, rs6602398, rs11256497, rs791587, rs10905669, rs2476491, rs2256774, rs706779, rs706778, rs3118470, *IL3* rs181781, *IL8RA* rs1008563, rs1008562, rs16858811, *IL8RB* rs1126579, *IRF2* rs809909, rs10009261, rs1425551, rs807684, rs3756094, *PRKAG2* rs1541538, rs2536082, rs6947064, rs7805747, rs1860743, rs10278273, rs7801616, rs7784818, rs3934597, *RAF1* rs3729931, rs9809501, rs11923427, rs4684871, rs904453, *RUNX3* rs7517302, rs2135756, *SEPX1* rs13331553, rs732510, *SOCS1* rs193779, *STK11* rs8111699, rs741765, *TSC2* rs2074968.

The polygenic risk score (Fig. 1) showed increased risk with increasing number of at risk alleles. The overall HR for colorectal cancer mortality among colon cancer cases in the highest risk group (upper sixth of the at-risk allele distribution) was 11.81 (95% CI 7.07, 19. 74) and was 10.99 (95% CI 5.30,22.78) among rectal cancer cases.

## Discussion

Several genes were associated with survival after diagnosis with colorectal cancer, although the overall pathway was not statistically significant and only the JAK/STAT/SOCs sub-pathway had a P_ARTP_<0.05. Fifteen genes were associated with colon cancer survival (P_ARTP_<0.05) and seven genes were associated with rectal cancer survival. It should be noted this represents 9.6% of genes analyzed for colon cancer and approximately 5% of genes analyzed for rectal cancer and could be chance findings; thus these findings need replications. We observed that the hazard of dying after being diagnosed with either colon or rectal cancer increased with increasing number of at-risk alleles. The lack of statistical significance observed for the overall pathway could reflect sub-pathway groupings that did not optimize the data. Further evaluation at the gene and SNP level suggested that many components of the pathway contributed to survival, although a large segment of the pathway did not.

The JAK/STAT-signaling pathway was the only sub-pathway that was statistically significant using ARTP. This pathway plays a critical role in immune response and regulation of inflammation given its essential affiliation with cytokine signaling. STAT3 specifically has been shown to promote uncontrolled cell growth and survival through dysregulation of gene expression involved in apoptosis, cell-cycle regulation, and angiogenesis. [18] *JAK1*, *JAK2*, and *STAT3* have been associated with colorectal cancer progression [19]. In our analysis, *STAT3* and *STAT5* were of marginal significance with colon cancer survival, while *JAK2* and *TYK2* were statistically significant. Within these genes, several SNPs were significantly associated with survival.

Several genes in the backbone of the CHIEF pathway were associated with survival, including *PIK3CA* for colon cancer and *PRKAG2*, *STK11*, and *TSC2* for rectal cancer. Phosphoinositide 3-kinase (PI3K gene official name *PIK3CA*) is an early event in cells responding to growth factors, cytokines, and insulin [20]. PI3K induces the activation of Akt1 (alias PDK). The PI3K/Akt pathway is recognized as an important regulator of cell proliferation and survival and is thought to be involved in mediating the effects of MTOR [21]. It has been shown that inflammation-related factors can activate MTOR can promote tumor angiogenesis by phosphorylating TSC1 (also known as hamartin) and thereby inactivating the TSC1-TSC2 complex [22], [23]. TSC2, also known as tuberin, specifically has been shown to be involved in insulin signaling, tumor suppressor functions, and regulation of cell growth. A study by Lee and colleagues showed that *STK11*, *PRKAA1*, and *TSC1* polymorphisms were associated with disease-free survival after diagnosis with colorectal cancer; they did not see an association with *TSC2* [24]. Other studies have shown that *STK11* is associated with tumor metastasis and more aggressive tumors [25], [26].

Increased tumor vascularization and inflammation have been associated with advanced tumor stage and poor prognosis [27]. Thus, we hypothesized that genes associated with angiogenesis would influence survival. We observed that *NOS2A*, *MMP1*, and *VDR* were associated with survival after colon cancer diagnosis and no major angiogenesis genes on our platform were associated with rectal cancer. Inducible nitric oxide synthase (NOS2) is induced by inflammatory cytokines and hypoxia and produces large amounts of nitric oxide. Nitric oxide can affect cancer through many ways, it can increase apoptosis and inhibit carcinogenesis or promote carcinogenesis through increasing angiogenesis [28]. MMPs are involved in normal physiological processes required for development and morphogenesis; a loss of control of MMPs can result in pathological processes including inflammation, angiogenesis, and cellular proliferation that are central to diseases such as cancer. MMPs, and MMP1 specifically, have been studied using indicators of metastatic potential by evaluating tumor stage at time of diagnosis, tumor grade and histology and been shown to be associated with greater metastatic potential [29]. VDR expression has been associated with better survival for colon and breast cancer [30]–[32]. Previously, we reported that *FLT1* SNPs were significantly associated with the hazard of dying of colorectal cancer after diagnosis with colon cancer and *KDR* SNPs were associated significantly with colorectal deaths after diagnosis with rectal cancer [33].

The TGF-β-signaling pathway has been shown to be one of the strongest pathways associated with colon cancer risk in our data. Others have shown that improved disease-free survival after diagnosis with CRC was associated with increased TGF-β expression [34]. Forsti and colleagues looked at nine polymorphisms in the TGF-β-signaling pathway and CRC among 308 cases of colorectal cancer [35] and observed that *TGFβRA* IVS7G+24A minor allele was associated with better survival. Several others studies have focused on *SMAD2*, *SMAD4*, and *SMAD7* and found associations with prognosis after CRC diagnosis [36], [37]. We only observed marginally significant associations with *BMP2* (P_ARTP_ = 0.083), *BMPR1A* (P_ARTP_ = 0.053), *BMPR1B* (P_ARTP_ = 0.069) for colon cancer survival. *RUNX3* was significantly associated with rectal cancer survival, while *BMP1* (P_ARTP_ = 0.099) and *BMPR1A* (P_ARTP_ = 0.085) were marginally significant.

Two *MAPKs* genes were associated with survival in our data; these genes mediate intracellular signaling and are involved in diverse cellular processes that include cell proliferation and differentiation and apoptosis and implicated in progression [38]. The three major categories of MAPK are the stress-activated protein kinase c-Jun NH-2 terminal kinase (*JNK* or *SAPK1*), stress-activated protein kinase 2 (p38 or SAPK2), and the extracellular signal-regulated protein kinases (*ERK1*/2) [38], [39]. JNK, which includes *MAP3K10* that was associated with survival in our data, is generally associated with apoptosis induction [40]. DUSPs attenuate the effect of MAPK [41].

*SEPX1* was associated with survival for both colon and rectal cancer while *SEP15* was marginally associated (P_ARTP_ = 0.068) with colon cancer survival. We previously reported that three SNPs in this pathway were associated with rectal cancer survival, *SEPN1* rs718391 (HR 1.67, 95% CI 1.11,2.51) and *SEPX1* rs13331553 (HR 1.46 95%CI 1.07,2.00) and *SEPX1* rs732510 (HR 1.68 95% CI 1.09,2.60) after adjustment for multiple comparisons using FDR. However, taking the gene approach as we did with ARTP, *SEPX1* remained significant for both colon and rectal cancer.

Several cytokines, including interleukins and interferons, and other mediators of inflammation were associated with both colon (*INFGR1*, *IL6*, *IRF2*, *NFκB1A*, *TLR2*) and rectal cancer survival (*IL1A* and *IL3*), as was suppressor of cytokine signaling (*SOCS1*). Functions of cytokine-related pathways include apoptosis and cell proliferation. INFG has been shown to regulate the expression of apoptosis-related genes and has been hypothesized to regulate cell sensitivity to apoptosis [42]. TLRs can promote inflammation, cell survival and tumor progression [43]. Studies analyzing associations between risk or survival and SNPs in interleukin genes such as *IL1B*, *IL1RA*, *IL10* have reported conflicting results; some SNPs being associated with increased risk or survival while others associated with a lower risk or survival for colorectal cancer [44]–[46].

To estimate the magnitude of risk associated with carrying multiple high-risk alleles, we created a polygenic risk score. Our results suggest that the genetic variant load is important for survival after diagnosis since we observed substantial increased risk of dying with increasing numbers of variant genotypes. While one could hypothesize that a single insult to the pathway could influence risk and that additional insults would have minimal effect on risk, our data suggest otherwise. Inflammatory pathways are somewhat redundant, composed of multiple cytokines with overlapping functions; this supports that multiple insults to the pathways would result in increased risk. Our data support the hypothesis that increases in risk and hazard of dying is linear and that as genetic variant load of high-risk genotypes increases, so does the risk of developing cancer and dying after being diagnosed with cancer. However, caution is in order given the data used to identify at-risk alleles, was then used in the polygenic risk score. While we did not just take significant SNPs in creating the risk score, but used our permutated data to identify at-risk alleles, these results still warrant caution, especially in terms of the magnitude of the associations detected. Furthermore, to help place the risk observed in these data to other risk factors for survival, it should be noted that disease stage remains the strongest predictor of survival, with those being diagnosed at AJCC Stage 4 having over a 12-fold increased risk of dying than those diagnosed at a local disease stage.

The pathway approach we used was novel in that it summarized the statistical significance of the pathway and genes rather than focus on individual SNPs. ARTP allowed us to combine single SNP p values using the rank truncated product statistic and assess significance via permutations at multiple levels, including the gene, sub-pathway, and overall pathway level. While we selected genes that we believed were most important to the pathway, there are many other genes and SNPs involved in this pathway that could be important and contribute to colorectal cancer-specific mortality. We also are limited in our ability to assess interaction between genes and with lifestyle factors that could influence risk, since ARTP at this time does not allow for assessment of interactions. Unfortunately, we do not have a separate population to validate these findings and therefore encourage others with similar data to replicate these findings. Likewise, we did not attempt a test and training set, given the impact of that method on study power; lack of replication thus could be from lack of power. Other limitations to our assessment is lack of treatment and other related medical conditions that could impact survival. While we can argue that it is unlikely that these genes and SNPs are associated with treatment, we do not have the ability to test that. However, treatment is highly correlated with AJCC stage, and we have adjusted for stage in our analysis.

It is noteworthy that our findings for colon and rectal cancer are for the most part different. There are several potential explanations for these findings. First, disease pathways could be different for the two cancer sites, and thus genes and sub-pathways that are important could also differ. Another explanation for these differences, could stem from a smaller sample size for rectal than colon cancer. This could explain the lack of replication in rectal cancer from colon cancer findings, however it would explain differences observed in rectal cancer that are not replicated in colon cancer. While the underlying cause of these differences is not clear, it has been observed that risk factors differ between colon and rectal cancer [11], [47]–[54].

In conclusion, there is support that genes within the CHIEF pathway are associated with colorectal cancer-specific mortality, although the overall pathway did not influence risk. Replication of these findings, along with more detailed assessment of the specific genes may help identify key variants that could importantly contribute to prognosis.

## Supporting Information

S1 Table **List of genes, aliases, and chromosomal location.**
(DOCX)

S2 Table **Table. List of sub-pathways and genes included in each sub-pathway for ARTP analysis.**
(DOCX)

S3 Table **Genes and related SNPs associated with colorectal cancer-specific mortality among patients diagnosed with colon cancer (0.05> gene P_ARTP_≤0.10; SNP P_trend_≤0.10).**
(DOCX)

S4 Table **Genes and related SNPs associated with colorectal cancer-specific mortality among patients diagnosed with rectal cancer (0.05> gene P_ARTP_≤0.10; SNP P_trend_≤0.10).**
(DOCX)
