# Supplementary material for: The Influence of the CHIEF Pathway on Colorectal Cancer-Specific Mortality
Source: PLoS One. 2014 Dec 26;9(12):e116169. doi: 10.1371/journal.pone.0116169 (PMC4277466; doi:10.1371/journal.pone.0116169)
Supplement: S1 Table — List of genes, aliases, and chromosomal location. (DOCX) [file pone.0116169.s001.docx]

| Supplemental Table S1. List of genes, aliases, and chromosomal location |  | |  | |  | |  |
| --- | --- | --- | --- | --- | --- | --- | --- |
| Official Gene Name | Common Aliases | | Chromosome | |  | |  |
| AKT1 (V-AKT MURINE THYMOMA VIRAL ONCOGENE HOMOLOG 1 ) | *AKT, MGC99656, PKB, PRKBA, RAC, RAC-ALPHA* | | 14q32.32 | |  | |  |
| AR (ANDROGEN RECEPTOR) | *DHTR, NR3C4* | | Xq12 | |  | |  |
| BMP1 (BONE MORPHOGENETIC PROTEIN 1 ) | *FLJ44432, PCOLC, PCP, TLD* | | 8p21 | |  | |  |
| BMP2 (BONE MORPHOGENETIC PROTEIN 2 ) | *BMP2A* | | 20p12 | |  | |  |
| BMP4 (BONE MORPHOGENETIC PROTEIN 4 ) | *BMP2B, BMP2B1, ZYME* | | 14q22-q23 | |  | |  |
| BMPR1A (BONE MORPHOGENETIC PROTEIN RECEPTOR, TYPE IA ) | *ACVRLK3, ALK3, CD292* | | 10q22.3 | |  | |  |
| BMPR1B (BONE MORPHOGENETIC PROTEIN RECEPTOR, TYPE IB ) | *ALK-6, ALK6, CDw293* | | 4q22-q24 | |  | |  |
| BMPR2 (BONE MORPHOGENETIC PROTEIN RECEPTOR, TYPE II) | *BMPR-II, BMPR3, BMR2* | | 2q33-q34 | |  | |  |
| C11orf31 (CHROMOSOME 11 OPEN READING FRAME 31 ) | *C17orf10, SELH* | | 11q12.1 | |  | |  |
| CYP19A1 (CYTOCHROME P450, FAMILY 19, SUBFAMILY A, POLYPEPTIDE 1 ) | *AROMATASE, CYP19* | | 15q21.1 | |  | |  |
| DUSP1 (DUAL SPECIFICITY PHOSPHATASE 1 ) | *MKP-1, MKP1, PTPN10* | | 5q34 | |  | |  |
| DUSP2 (DUAL SPECIFICITY PHOSPHATASE 2 ) | *PAC-1, PAC1* | | 2q11 | |  | |  |
| DUSP4 (DUAL SPECIFICITY PHOSPHATASE 4 ) | *HVH2, MKP-2, MKP2, TYP* | | 8p12-p11 | |  | |  |
| DUSP6 (DUAL SPECIFICITY PHOSPHATASE 6 ) | *MKP3, PYST1* | | 12q22-q23 | |  | |  |
| DUSP7 (DUAL SPECIFICITY PHOSPHATASE 7 ) | *MKP-X, MKPX, PYST2* | | 3p21 | |  | |  |
| EGFR (EPIDERMAL GROWTH FACTOR RECEPTOR) | *ERBB, ERBB1, HER1* | | 7p12 | |  | |  |
| EGR2 (EARLY GROWTH RESPONSE 2 ) | *CMT1D, CMT4E, KROX20* | | 10q21.1 | |  | |  |
| EIF4E (EUKARYOTIC TRANSLATION INITIATION FACTOR 4E ) | *CBP, EIF4E1, EIF4EL1, EIF4F* | | 4q21-q25 | |  | |  |
| EIF4EBP2 (EUKARYOTIC TRANSLATION INITIATION FACTOR 4E BINDING PROTEIN 2 ) | *4EBP2* | | 10q21-q22 | |  | |  |
| EIF4EBP3 (EUKARYOTIC TRANSLATION INITIATION FACTOR 4E BINDING PROTEIN 3 ) | *4E-BP3* | | 5q31.3 | |  | |  |
| EPX (EOSINOPHIL PEROXIDASE ) | *EPO, EPP, EPX-PEN* | | 17q23.1 | |  | |  |
| ESR1 (ESTROGEN RECEPTOR 1) | *ESR, ER, ESRA* | | 6q21.5 | |  | |  |
| ESR2 (ESTROGEN RECEPTOR 2) | *ESRB, ESR-Beta, ER-Beta* | | 14q23.2-q23.3 | |  | |  |
| FLT1 (FMS-RELATED TYROSINE KINASE 1) | *FLT, VEGFR1* | | 13q12 | |  | |  |
| FRAP1 (FK506 BINDING PROTEIN 12-RAPAMYCIN ASSOCIATED PROTEIN 1 ) | *FRAP, FRAP2, MTOR, RAFT1, RAPT1* | | 1p36.2 | |  | |  |
| GDF10 (GROWTH DIFFERENTIATION FACTOR 10 ) | *BMP-3b, BMP3B* | | 10q11.22 | |  | |  |
| HIF1A (HYPOXIA-INDUCIBLE FACTOR 1, ALPHA SUBUNIT) | *HIF-1alpha, HIF1, HIF1-ALPHA, MOP1* | | 14q21-q24 | |  | |  |
| IFNG (INTERFERON, GAMMA ) | *IFG, IFI* | | 12q14 | |  | |  |
| IFNGR1 (INTERFERON GAMMA RECEPTOR 1 ) | *CD119, FLJ45734, IFNGR* | | 6q23-q24 | |  | |  |
| IFNGR2 (INTERFERON GAMMA RECEPTOR 2) | *AF-1, IFGR2, IFNGT1* | | 21q22.11 | |  | |  |
| IGF1 (INSULIN-LIKE GROWTH FACTOR I) | *IGF I* | | 12q23.2 | |  | |  |
| IGF1R (INSULIN-LIKE GROWTH FACTOR RECEPTOR I) |  | | 15q26.3 | |  | |  |
| IGFPB3 (INSULIN-LIKE GROWTH FACTOR-BINDING PROTEIN 3) | *IBP3* | | 7p12.3 | |  | |  |
| IKBKB (INHIBITOR OF KAPPA LIGHT POLYPEPTIDE GENE ENHANCER IN B-CELLS) | *FLJ40509, IKK-beta, IKK2, IKKB, NFKBIKB* | | 8p11.2 | |  | |  |
| IL10 (INTERLEUKIN 10 ) | *CSIF, IL-10, IL10A, TGIF* | | 1q31-q32 | |  | |  |
| IL15 (INTERLEUKIN 15 ) | *IL-15, MGC9721* | | 4q31 | |  | |  |
| IL17A (INTERLEUKIN 17A ) | *CTLA8, IL-17, IL-17A, IL17* | | 6p12 | |  | |  |
| IL1A (INTERLEUKIN 1, ALPHA ) | *IL-1A, IL1, IL1-ALPHA, IL1F1* | | 2q14 | |  | |  |
| IL1B (INTERLEUKIN 1, BETA ) | *IL-1, IL1-BETA, IL1F2* | | 2q14 | |  | |  |
| IL1RN (INTERLEUKIN 1 RECEPTOR ANTAGONIST ) | *ICIL-1RA, IL-1ra3, IL1F3, IL1RA, IRAP* | | 2q14.2 | |  | |  |
| IL2 (INTERLEUKIN 2 ) | *IL-2, TCGF, lymphokine* | | 4q26-q27 | |  | |  |
| IL23R (INTERLEUKIN 23 RECEPTOR ) |  | | 1p31.3 | |  | |  |
| IL2RA (INTERLEUKIN 2 RECEPTOR, ALPHA ) | *CD25, IDDM10, IL2R, TCGFR* | | 10p15-p14 | |  | |  |
| IL3 (INTERLEUKIN 3) | *IL-3, MULTI-CSF* | | 5q31.1 | |  | |  |
| IL4 (INTERLEUKIN 4 ) | *BSF1, IL-4* | | 5q31.1 | |  | |  |
| IL6 (INTERLEUKIN 6 (INTERFERON, BETA 2) ) | *BSF2, HGF, HSF, IFNB2, IL-6* | | 7p21 | |  | |  |
| IL6R (INTERLEUKIN 6 RECEPTOR ) | *CD126, IL-6R-1, IL-6R-alpha, IL6RA* | | 1q21 | |  | |  |
| IL8 (INTERLEUKIN 8 ) | *AMCF-I, CXCL8, GCP-1, GCP1, K60, NAF, NAP1* | | 4q13-q21 | |  | |  |
| IL8RA (INTERLEUKIN 8 RECEPTOR, ALPHA ) | *CD128, CD181, CDw128a, CKR-1, IL8R1, IL8RBA* | | 2q35 | |  | |  |
| IL8RB (INTERLEUKIN 8 RECEPTOR, BETA ) | *CD182, CDw128b, CXCR2, IL8R2, IL8RA* | | 2q35 | |  | |  |
| IRF1 (INTERFERON REGULATORY FACTOR 1 ) | *IRF-1, MAR* | | 5q31.1 | |  | |  |
| IRF2 (INTERFERON REGULATORY FACTOR 2 ) | *DKFZp686F0244, IRF-2* | | 4q34.1-q35.1 | |  | |  |
| IRF3 (INTERFERON REGULATORY FACTOR 3 ) |  | | 19q13.3-q13.4 | |  | |  |
| IRF4 (INTERFERON REGULATORY FACTOR 4 ) | *LSIRF, MUM1* | | 6p25-p23 | |  | |  |
| IRF5 (INTERFERON REGULATORY FACTOR 5 ) |  | | 7q32 | |  | |  |
| IRF6 (INTERFERON REGULATORY FACTOR 6 ) | *LPS, OFC6, PIT, PPS, VWS* | | 1q32.3-q41 | |  | |  |
| IRF7 (INTERFERON REGULATORY FACTOR 7 ) | *IRF-7H, IRF7A* | | 11p15.5 | |  | |  |
| IRF8 (INTERFERON REGULATORY FACTOR 8 ) | *H-ICSBP, ICSBP, ICSBP1, IRF-8* | | 16q24.1 | |  | |  |
| IRF9 (INTERFERON REGULATORY FACTOR 9 ) | *IRF-9, ISGF3, ISGF3G, p48* | | 14q11.2 | |  | |  |
| IRGM (IMMUNITY-RELATED GTPASE FAMILY, M ) | *IFI1, IRGM1, LRG-47, LRG47* | | 5q33.1 | |  | |  |
| IRS1 (INSULIN RECEPTOR SUBSTRATE 1) |  | | 2q36.3 | |  | |  |
| IRS2 (INSULIN RECEPTOR SUBSTRATE 2) |  | | 13q34 | |  | |  |
| JAK1 (JANUS KINASE 1 (A PROTEIN TYROSINE KINASE) ) | *JAK1A, JAK1B* | | 1p32.3-p31.3 | |  | |  |
| JAK2 (JANUS KINASE 2 (A PROTEIN TYROSINE KINASE) ) |  | | 9p24 | |  | |  |
| JUN (JUN ONCOGENE ) | *AP1, c-Jun* | | 1p32-p31 | |  | |  |
| JUNB (JUN B PROTO-ONCOGENE ) |  | | 19p13.2 | |  | |  |
| KDR (KINASE INSERT DOMAIN RECEPTOR) | *CD309, FLK1, VEGFR, VEGFR2* | | 4q11-q12 | |  | |  |
| MAP2K1 (MITOGEN-ACTIVATED PROTEIN KINASE KINASE 1 ) | *MAPKK1, MEK1, MKK1, PRKMK1* | | 15q22.1-q22.33 | |  | |  |
| MAP3K1 (MITOGEN-ACTIVATED PROTEIN KINASE KINASE KINASE 1 ) | *MAPKKK1, MEKK, MEKK1* | | 5q11.2 | |  | |  |
| MAP3K10 (MITOGEN-ACTIVATED PROTEIN KINASE KINASE KINASE 10 ) | *MLK2, MST* | | 19q13.2 | |  | |  |
| MAP3K11 (MITOGEN-ACTIVATED PROTEIN KINASE KINASE KINASE 11 ) | *MGC17114, MLK-3, MLK3, PTK1, SPRK* | | 11q13.1-q13.3 | |  | |  |
| MAP3K2 (MITOGEN-ACTIVATED PROTEIN KINASE KINASE KINASE 2 ) | *MEKK2, MEKK2B* | | 2q14.3 | |  | |  |
| MAP3K3 (MITOGEN-ACTIVATED PROTEIN KINASE KINASE KINASE 3 ) | *MAPKKK3, MEKK3* | | 17q23.3 | |  | |  |
| MAP3K7 (MITOGEN-ACTIVATED PROTEIN KINASE KINASE KINASE 7 ) | *TAK1, TGF1a* | | 6q16.1-q16.3 | |  | |  |
| MAP3K9 (MITOGEN-ACTIVATED PROTEIN KINASE KINASE KINASE 9 ) | *MLK1, PRKE1* | | 14q24.3-q31 | |  | |  |
| MAPK1 (MITOGEN-ACTIVATED PROTEIN KINASE 1 ) | *ERK, ERK2, ERT1, MAPK2, P42MAPK, PRKM1, PRKM2, p38p40, p41, p41mapk* | | 22q11.21 | |  | |  |
| MAPK12 (MITOGEN-ACTIVATED PROTEIN KINASE 12 ) | *ERK3, ERK6, P38GAMMA, PRKM12, SAPK-3, SAPK3* | | 22q13.33 | |  | |  |
| MAPK14 (MITOGEN-ACTIVATED PROTEIN KINASE 14 ) | *CSBP1, CSBP2, CSPB1, PRKM14, PRKM15RK, SAPK2A, p38, p38ALPHA* | | 6p21.3-p21.2 | |  | |  |
| MAPK3 (MITOGEN-ACTIVATED PROTEIN KINASE 3 ) | *ERK1, P44ERK1, P44MAPK, PRKM3* | | 16p11.2 | |  | |  |
| MAPK8 (MITOGEN-ACTIVATED PROTEIN KINASE 8 ) | *JNK, JNK1, JNK1A2, JNK21B1/2, PRKM8, SAPK1* | | 10q11.22 | |  | |  |
| MMP1 (MATRIX METALLOPEPTIDASE 1) | *CLG, CLGN* | | 11q22.3 | |  | |  |
| MMP3 (MATRIX METALLOPEPTIDASE 3) | *CHDS6, MMP-3, STMY, STMY1STR1* | | 11q22.3 | |  | |  |
| MMP7 (MATRIX METALLOPEPTIDASE 7) | *MMP-7, MPSL1, PUMP-1* | | 11q21-q22 | |  | |  |
| MMP9 (MATRIX METALLOPEPTIDASE 9) | *CLG4B, GELB, MANDP2, MMP-9* | | 20q11.2-q13.1 | |  | |  |
| MPO (MYELOPEROXIDASE ) |  | | 17q23.1 | |  | |  |
| MSTN (MYOSTATIN ) | *GDF8* | | 2q32.2 | |  | |  |
| NFAM1 (NFAT ACTIVATING PROTEIN WITH ITAM MOTIF 1 ) | *CNAIP, FLJ40652* | | 22q13.2 | |  | |  |
| NFAT5 (NUCLEAR FACTOR OF ACTIVATED T-CELLS 5) | *NF-AT5, NFATL1, NFATZ* | | 16q22.1 | |  | |  |
| NFKB1 (NUCLEAR FACTOR OF KAPPA LIGHT POLYPEPTIDE GENE ENHANCER IN B-CELLS 1 (P105) ) | *EBP-1, KBF1, MGC54151, NF-kappa-B, NFKB-p105, NFKB-p50* | | 4q24 | |  | |  |
| NFKB2 (NUCLEAR FACTOR OF KAPPA LIGHT POLYPEPTIDE GENE ENHANCER IN B-CELLS 2 (P49/P100) ) | *LYT-10, LYT10* | | 10q24 | |  | |  |
| NFKBIA (NUCLEAR FACTOR OF KAPPA LIGHT POLYPEPTIDE GENE ENHANCER IN B-CELLS INHIBITOR, ALPHA ) | *IKBA, MAD-3, NFKBI* | | 14q13 | |  | |  |
| NOS2A (NITRIC OXIDE SYNTHASE 2A (INDUCIBLE, HEPATOCYTES) ) | *HEP-NOS, INOS, NOS, NOS2* | | 17q11.2-q12 | |  | |  |
| PDGFB (PLATELET-DERIVED GROWTH FACTOR BETA POLYPEPTIDE) | *PDGF2, SIS* | | 22q13.1 | |  | |  |
| PDK1 (PYRUVATE DEHYDROGENASE KINASE, ISOZYME 1 ) |  | | 2q31.1 | |  | |  |
| PDK2 (PYRUVATE DEHYDROGENASE KINASE, ISOZYME 2 ) |  | | 17q21.33 | |  | |  |
| PIK3CA (PHOSPHOINOSITIDE-3-KINASE, CATALYTIC, ALPHA POLYPEPTIDE ) | *PI3K, p110-alpha* | | 3q26.3 | |  | |  |
| PIK3CB (PHOSPHOINOSITIDE-3-KINASE, CATALYTIC, BETA POLYPEPTIDE ) | *PI3K, PI3Kbeta, PIK3C1, p110-BETA* | | 3q22.3 | |  | |  |
| PIK3CG (PHOSPHOINOSITIDE-3-KINASE, CATALYTIC, GAMMA POLYPEPTIDE ) | *PI3CG, PI3K, PI3Kgamma, PIK3* | | 7q22.3 | |  | |  |
| PPARG (PEROXISOME PROLIFERATOR-ACTIVATED RECEPTOR-GAMMA) |  | | 3p25.2 | |  | |  |
| PRKAA1 (PROTEIN KINASE, AMP-ACTIVATED, ALPHA 1 CATALYTIC SUBUNIT ) | *AMPK, AMPKa1* | | 5p12 | |  | |  |
| PRKAA2 (PROTEIN KINASE, AMP-ACTIVATED, ALPHA 2 CATALYTIC SUBUNIT ) | *AMPK, AMPK2, PRKAA* | | 1p31 | |  | |  |
| PRKAB1 (PROTEIN KINASE, AMP-ACTIVATED, BETA 1 NON-CATALYTIC SUBUNIT ) | *AMPK, HAMPKb* | | 12q24.1 | |  | |  |
| PRKAB2 (PROTEIN KINASE, AMP-ACTIVATED, BETA 2 NON-CATALYTIC SUBUNIT ) | *MGC61468* | | 1q21.1 | |  | |  |
| PRKAG2 (PROTEIN KINASE, AMP-ACTIVATED, GAMMA 2 NON-CATALYTIC SUBUNIT ) | *AAKG, AAKG2, CMH6, WPWS* | | 7q36.1 | |  | |  |
| PTEN (PHOSPHATASE AND TENSIN HOMOLOG) | *BZS, MMAC1, PTEN1, TEP1* | | 10q23.3 | |  | |  |
| RAF1 (V-RAF-1 MURINE LEUKEMIA VIRAL ONCOGENE HOMOLOG 1 ) | *CRAF, Raf-1, c-Raf* | | 3p25 | |  | |  |
| RPS6KA1 (RIBOSOMAL PROTEIN S6 KINASE, 90KDA, POLYPEPTIDE 1 ) | *HU-1, MAPKAPK1A, RSK, RSK1, S6K-alpha1* | | 1p | |  | |  |
| RPS6KA2 (RIBOSOMAL PROTEIN S6 KINASE, 90KDA, POLYPEPTIDE 2 ) | *MAPKAPK1C, RSK, RSK3, S6K-alpha, S6K-alpha2, p90-RSK3* | | 6q27 | |  | |  |
| RPS6KB1 (RIBOSOMAL PROTEIN S6 KINASE, 70KDA, POLYPEPTIDE 1 ) | *PS6K, S6K, S6K1, STK14A, p70(S6K)-alpha, p70-S6K* | | 17q23.1 | |  | |  |
| RPS6KB2 (RIBOSOMAL PROTEIN S6 KINASE, 70KDA, POLYPEPTIDE 2 ) | *P70-beta, S6K-beta2, S6K2, SRK, STK14Bp70(S6K)-beta, p70S6Kb* | | 11q13.2 | |  | |  |
| RUNX1 (RUNT-RELATED TRANSCRIPTION FACTOR 1) | *AML1, AML1-EVI-1, AMLCR1, PEBP2aB* | | 21q22.3 | |  | |  |
| RUNX2 (RUNT-RELATED TRANSCRIPTION FACTOR 2 ) | *AML3, CBFA1, CCD, CCD1, PEBP2A1, PEBP2A2, PEBP2aA, PEBP2aA1* | | 6p21 | |  | |  |
| RUNX3 (RUNT-RELATED TRANSCRIPTION FACTOR 3 ) | *AML2, CBFA3,PEBP2aC* | | 1p36 | |  | |  |
| SELS (SELENOPROTEIN S ) | *ADO15, SBBI8, SEPS1, VIMP* | | 15q26.3 | |  | |  |
| SEP15 (15 KDA SELENOPROTEIN ) |  | | 1p31 | |  | |  |
| SEPN1 (SELENOPROTEIN N, 1 ) | *MDRS1, RSMD1, RSS, SELN* | | 1p36.13 | |  | |  |
| SEPP1 (SELENOPROTEIN P, PLASMA, 1 ) | *SELP, SeP* | | 5q31 | |  | |  |
| SEPW1 (SELENOPROTEIN W, 1 ) | *selW* | | 19q13.3 | |  | |  |
| SEPX1 (SELENOPROTEIN X, 1 ) | *MSRB1, SELR, SELX* | | 16p13.3 | |  | |  |
| SLC2A4 (SOLUTE CARRIER FAMILY 2, MEMBER 4 ) | *GLUT4* | | 17p13 | |  | |  |
| SMAD1 (SMAD FAMILY MEMBER 1 ) | *MADH1, MADR1* | | 4q31 | |  | |  |
| SMAD2 (SMAD FAMILY MEMBER 2 ) | *MADH2, MADR2, hSMAD2* | | 18q21.1 | |  | |  |
| SMAD3 (SMAD FAMILY MEMBER 3 ) | *MADH3, MGC60396, Smad3* | | 15q22.33 | |  | |  |
| SMAD4 (SMAD FAMILY MEMBER 4 ) | *DPC4, JIP, MADH4* | | 18q21.1 | |  | |  |
| SMAD7 (SMAD FAMILY MEMBER 7 ) | *FLJ16482, MADH7, MADH8* | | 18q21.1 | |  | |  |
| SOCS1 (SUPPRESSOR OF CYTOKINE SIGNALING 1 ) | *CIS1, CISH1, JAB, SOCS-1, SSI-1, SSI1, TIP3* | | 16p13.13 | |  | |  |
| SOCS2 (SUPPRESSOR OF CYTOKINE SIGNALING 2 ) | *CIS2, Cish2, SOCS-2, SSI-2, SSI2, STATI2* | | 12q | |  | |  |
| SOD1 (SUPEROXIDE DISMUTASE 1) | *IPOA* | | 21q22.11 | |  | |  |
| SOD2 (SUPEROXIDE DISMUTASE 2) | *IPO-B, MNSOD* | | 6q25.11 | |  | |  |
| STAT1 (SIGNAL TRANSDUCER AND ACTIVATOR OF TRANSCRIPTION 1, 91KDA ) | *ISGF-3, STAT91* | | 2q32.2 | |  | |  |
| STAT2 (SIGNAL TRANSDUCER AND ACTIVATOR OF TRANSCRIPTION 2, 113KDA ) | *ISGF-3, P113, STAT113* | | 12q13.2 | |  | |  |
| *STAT3 (SIGNAL TRANSDUCER AND ACTIVATOR OF TRANSCRIPTION 3)* | | *APRF* | | 17q21.31 | |  | |
| STAT4 (SIGNAL TRANSDUCER AND ACTIVATOR OF TRANSCRIPTION 4 ) |  | | 2q32.2-q32.3 | |  | |  |
| STAT5A (SIGNAL TRANSDUCER AND ACTIVATOR OF TRANSCRIPTION 5A ) | *MGF, STAT5* | | 17q11.2 | |  | |  |
| STAT5B (SIGNAL TRANSDUCER AND ACTIVATOR OF TRANSCRIPTION 5B ) | *STAT5* | | 17q11.2 | |  | |  |
| STAT6 (SIGNAL TRANSDUCER AND ACTIVATOR OF TRANSCRIPTION 6, INTERLEUKIN-4 INDUCED ) | *IL-4-STAT, STAT6B, STAT6C* | | 12q13 | |  | |  |
| STK11 (SERINE/THREONINE KINASE 11 ) | *LKB1, PJS* | | 19p13.3 | |  | |  |
| TCF7L2 (TRANSCRIPTION FACTOR 7-LIKE 2) | *TCF4* | | 10q25.2-q25.3 | |  | |  |
| TERT (TELOMERASE REVERSE TRANSCRIPTASE ) | *EST2, TCS1, TP2, TRT, hEST2* | | 5p15.33 | |  | |  |
| TGFB1 (TRANSFORMING GROWTH FACTOR, BETA 1 ) | *CED, DPD1, LAP, TGFB, TGFbeta* | | 19q13.1 | |  | |  |
| TGFBR1 (TRANSFORMING GROWTH FACTOR, BETA RECEPTOR 1 ) | *AAT5, ACVRLK4, ALK-5, ALK5, LDS1A, LDS2A, SKR4, TGFR-1* | | 9q22 | |  | |  |
| TLR2 (TOLL-LIKE RECEPTOR 2 ) | *CD282, TIL4* | | 4q32 | |  | |  |
| TLR3 (TOLL-LIKE RECEPTOR 3 ) | *CD283* | | 4q35 | |  | |  |
| TLR4 (TOLL-LIKE RECEPTOR 4 ) | *ARMD10, CD284, TOLL, hToll* | | 9q32-q33 | |  | |  |
| TNF (TUMOR NECROSIS FACTOR (TNF SUPERFAMILY, MEMBER 2) ) | *DASS-280D8.2, DIF, TNF-alpha, TNFA, TNFSF2* | | 6p21.3 | |  | |  |
| TNFRSF1A (TUMOR NECROSIS FACTOR RECEPTOR SUPERFAMILY, MEMBER 1A ) | *CD120a, TNF-R, TNF-R-I, TNFR55, TNFR60, p55, p55-R, p60* | | 12p13.2 | |  | |  |
| TRAF2 (TNF RECEPTOR-ASSOCIATED FACTOR 2 ) | *MGC* | | 9q34 | |  | |  |
| TSC1 (TUBEROUS SCLEROSIS 1 ) | *KIAA0243, LAM, MGC86987, TSC* | | 9q34 | |  | |  |
| TSC2 (TUBEROUS SCLEROSIS 2 ) | *FLJ43106, LAM, TSC4* | | 16p13.3 | |  | |  |
| TXNRD1 (THIOREDOXIN REDUCTASE 1 ) | *GRIM-12, MGC9145, TR, TR1, TRXR1, TXNR* | | 12q23-q24.1 | |  | |  |
| TXNRD2 (THIOREDOXIN REDUCTASE 2 ) | *SELZ, TR, TR-BETA, TR3, TRXR2* | | 22q11.21 | |  | |  |
| TXNRD3 (THIOREDOXIN REDUCTASE 3 ) | *TGR, TR2, TRXR3* | | 3q21.3 | |  | |  |
| TYK2 (TYROSINE KINASE 2 ) | *JTK1* | | 19p13.2 | |  | |  |
| VDR (VITAMIN D RECEPTOR) |  | | 12q13.11 | |  | |  |
| VEGFA (VASCULAR ENDOTHELIAL GROWTH FACTOR A ) | *MGC70609, VEGF, VEGF-A, VPF* | | 6p12 | |  | |  |
|  |  | |  | |  | |  |
