# Supplementary material for: The Influence of the CHIEF Pathway on Colorectal Cancer-Specific Mortality
Source: PLoS One. 2014 Dec 26;9(12):e116169. doi: 10.1371/journal.pone.0116169 (PMC4277466; doi:10.1371/journal.pone.0116169)
Supplement: S2 Table — Table. List of sub-pathways and genes included in each sub-pathway for ARTP analysis. (DOCX) [file pone.0116169.s002.docx]

Supplemental Table S2. List of sub-pathways and genes included in each sub-pathway for ARTP analysis.

|  |  |  | # SNPs /  Sub-pathway | P_ARTP_ Colon Cancer | P_ARTP_ Rectal Cancer |
| --- | --- | --- | --- | --- | --- |
| Sub-pathway | Gene | # SNPs |  |  |  |
| Angiogenesis | *EPX* | 9 | 114 | 0.6504 | 0.9516 |
| Angiogenesis | *FLT1* | 38 |  | 0.3915 | 0.8691 |
| Angiogenesis | *HIF1A* | 4 |  | 0.9508 | 0.4239 |
| Angiogenesis | *KDR* | 22 |  | 0.2795 | 0.1296 |
| Angiogenesis | *MMP1* | 1 |  | 0.0289 | 0.8161 |
| Angiogenesis | *MMP3* | 1 |  | 0.0693 | 0.2350 |
| Angiogenesis | *MMP7* | 1 |  | 0.3338 | 0.2136 |
| Angiogenesis | *MMP9* | 1 |  | 0.6859 | 0.8742 |
| Angiogenesis | *MPO* | 2 |  | 0.4102 | 0.3236 |
| Angiogenesis | *MSTN* | 2 |  | 0.7655 | 0.3586 |
| Angiogenesis | *NOS2A* | 16 |  | 0.0421 | 0.6877 |
| Angiogenesis | *SOD1* | 1 |  | 0.8612 | 0.8794 |
| Angiogenesis | *SOD2* | 5 |  | 0.7542 | 0.5197 |
| Angiogenesis | *VEGFA* | 11 |  | 0.3953 | 0.4759 |
| Hormone/Insulin/Growth | *AR* | 1 | 99 | 0.8604 | 0.5947 |
| Hormone/Insulin/Growth | *CYP19A1* | 27 |  | 0.4830 | 0.3108 |
| Hormone/Insulin/Growth | *EGFR* | 37 |  | 0.8679 | 0.9747 |
| Hormone/Insulin/Growth | *EGR2* | 4 |  | 0.6027 | 0.1533 |
| Hormone/Insulin/Growth | *ESR1* | 1 |  | 0.1459 | 0.6755 |
| Hormone/Insulin/Growth | *ESR2* | 2 |  | 0.3307 | 0.0857 |
| Hormone/Insulin/Growth | *IGF1* | 2 |  | 0.0611 | 0.8542 |
| Hormone/Insulin/Growth | *IGF1R* | 1 |  | 0.3616 | 0.8252 |
| Hormone/Insulin/Growth | *IGFPB3* | 2 |  | 0.9516 | 0.6935 |
| Hormone/Insulin/Growth | *IRS1* | 1 |  | 0.1523 | 0.9903 |
| Hormone/Insulin/Growth | *IRS2* | 1 |  | 0.1192 | 0.1238 |
| Hormone/Insulin/Growth | *PDGFB* | 9 |  | 0.4454 | 0.2771 |
| Hormone/Insulin/Growth | *PPARG* | 1 |  | 0.8830 | 0.8894 |
| Hormone/Insulin/Growth | *SLC2A4* | 5 |  | 0.5395 | 0.8742 |
| Hormone/Insulin/Growth | *TCF7L2* | 1 |  | 0.5979 | not available |
| Hormone/Insulin/Growth | *VDR* | 4 |  | 0.0499 | 0.7504 |
| Interferon | *IFNG* | 3 | 116 | 0.2059 | 0.2010 |
| Interferon | *IFNGR1* | 4 |  | 0.0121 | 0.6298 |
| Interferon | *IFNGR2* | 5 |  | 0.1860 | 0.1367 |
| Interferon | *IRF1* | 2 |  | 0.4163 | 0.5327 |
| Interferon | *IRF2* | 54 |  | 0.0207 | 0.0091 |
| Interferon | *IRF3* | 2 |  | 0.8505 | 0.8826 |
| Interferon | *IRF4* | 10 |  | 0.6047 | 0.5085 |
| Interferon | *IRF5* | 4 |  | 0.8040 | 0.8247 |
| Interferon | *IRF6* | 6 |  | 0.2149 | 0.6174 |
| Interferon | *IRF7* | 4 |  | 0.4961 | 0.9374 |
| Interferon | *IRF8* | 17 |  | 0.0675 | 0.8536 |
| Interferon | *IRF9* | 2 |  | 0.6846 | 0.9015 |
| Interferon | *IRGM* | 3 |  | 0.1265 | 0.4613 |
| Interleukin | *IL10* | 9 | 116 | 0.6467 | 0.7380 |
| Interleukin | *IL15* | 6 |  | 0.3102 | 0.8333 |
| Interleukin | *IL17A* | 1 |  | 0.6290 | 0.6170 |
| Interleukin | *IL1A* | 5 |  | 0.2364 | 0.0537 |
| Interleukin | *IL1B* | 5 |  | 0.0714 | 0.7797 |
| Interleukin | *IL1RN* | 13 |  | 0.1824 | 0.8047 |
| Interleukin | *IL2* | 4 |  | 0.5732 | 0.7753 |
| Interleukin | *IL23R* | 18 |  | 0.1726 | 0.3495 |
| Interleukin | *IL2RA* | 25 |  | 0.2474 | 0.0216 |
| Interleukin | *IL3* | 2 |  | 0.4813 | 0.0965 |
| Interleukin | *IL4* | 5 |  | 0.2547 | 0.2938 |
| Interleukin | *IL6* | 6 |  | 0.0417 | 0.4048 |
| Interleukin | *IL6R* | 8 |  | 0.3773 | 0.6203 |
| Interleukin | *IL8* | 3 |  | 0.1571 | 0.8173 |
| Interleukin | *IL8RA* | 4 |  | 0.1720 | 0.0189 |
| Interleukin | *IL8RB* | 2 |  | 0.9977 | 0.0306 |
| Jak/Stat/Socs | *JAK1* | 10 | 90 | 0.7236 | 0.5576 |
| Jak/Stat/Socs | *JAK2* | 10 |  | 0.0086 | 0.8521 |
| Jak/Stat/Socs | *SOCS1* | 2 |  | 0.0821 | 0.0965 |
| Jak/Stat/Socs | *SOCS2* | 4 |  | 0.4156 | 0.3952 |
| Jak/Stat/Socs | *STAT1* | 19 |  | 0.9568 | 0.1461 |
| Jak/Stat/Socs | *STAT2* | 3 |  | 0.7693 | 0.8173 |
| Jak/Stat/Socs | *STAT3* | 6 |  | 0.0543 | 0.9723 |
| Jak/Stat/Socs | *STAT4* | 21 |  | 0.8576 | 0.1775 |
| Jak/Stat/Socs | *STAT5A* | 2 |  | 0.0914 | 0.2631 |
| Jak/Stat/Socs | *STAT5B* | 3 |  | 0.2484 | 0.4554 |
| Jak/Stat/Socs | *STAT6* | 5 |  | 0.4619 | 0.6611 |
| Jak/Stat/Socs | *TYK2* | 5 |  | 0.0178 | 0.1321 |
| MAPK | *DUSP1* | 2 | 99 | 0.5981 | 0.7860 |
| MAPK | *DUSP2* | 1 |  | 0.0225 | 0.1436 |
| MAPK | *DUSP4* | 6 |  | 0.4792 | 0.2222 |
| MAPK | *DUSP6* | 2 |  | 0.9720 | 0.8657 |
| MAPK | *DUSP7* | 1 |  | 0.5407 | 0.8862 |
| MAPK | *MAP2K1* | 7 |  | 0.3570 | 0.3979 |
| MAPK | *MAP3K1* | 8 |  | 0.1564 | 0.8994 |
| MAPK | *MAP3K10* | 3 |  | 0.0306 | 0.7619 |
| MAPK | *MAP3K11* | 4 |  | 0.2015 | 0.1207 |
| MAPK | *MAP3K2* | 3 |  | 0.8325 | 0.5395 |
| MAPK | *MAP3K3* | 3 |  | 0.7096 | 0.7019 |
| MAPK | *MAP3K7* | 6 |  | 0.9178 | 0.4368 |
| MAPK | *MAP3K9* | 19 |  | 0.4014 | 0.6639 |
| MAPK | *MAPK1* | 6 |  | 0.4335 | 0.4828 |
| MAPK | *MAPK12* | 2 |  | 0.9328 | 0.9041 |
| MAPK | *MAPK14* | 12 |  | 0.4776 | 0.5896 |
| MAPK | *MAPK3* | 1 |  | 0.5812 | 0.8936 |
| MAPK | *MAPK8* | 5 |  | 0.4188 | 0.1364 |
| MAPK | *RAF1* | 8 |  | 0.5949 | 0.0158 |
| Pathway Core | *AKT1* | 2 | 287 | 0.4439 | 0.6083 |
| Pathway Core | *mTOR* | 4 |  | 0.3092 | 0.2079 |
| Pathway Core | *IKBKB* | 5 |  | 0.0899 | 0.3948 |
| Pathway Core | *JUN* | 1 |  | 0.9662 | 0.5315 |
| Pathway Core | *JUNB* | 1 |  | 0.0703 | 0.8376 |
| Pathway Core | *NFKB1* | 18 |  | 0.3587 | 0.7428 |
| Pathway Core | *NFKB2* | 2 |  | 0.6052 | 0.3771 |
| Pathway Core | *NFKBIA* | 3 |  | 0.0252 | 0.3078 |
| Pathway Core | *PDK1* | 3 |  | 0.3574 | 0.4231 |
| Pathway Core | *PDK2* | 3 |  | 0.4611 | 0.8645 |
| Pathway Core | *PIK3CA* | 7 |  | 0.0098 | 0.3269 |
| Pathway Core | *PIK3CB* | 2 |  | 0.3317 | 0.5373 |
| Pathway Core | *PIK3CG* | 10 |  | 0.3819 | 0.3196 |
| Pathway Core | *PRKAA1* | 13 |  | 0.1324 | 0.3004 |
| Pathway Core | *PRKAA2* | 9 |  | 0.3801 | 0.3210 |
| Pathway Core | *PRKAB1* | 4 |  | 0.1144 | 0.2953 |
| Pathway Core | *PRKAB2* | 8 |  | 0.5727 | 0.5903 |
| Pathway Core | *PRKAG2* | 69 |  | 0.1773 | 0.0817 |
| Pathway Core | *PTEN* | 9 |  | 0.9131 | 0.8528 |
| Pathway Core | *RPS6KA1* | 10 |  | 0.2322 | 0.1113 |
| Pathway Core | *RPS6KA2* | 71 |  | 0.0618 | 0.3102 |
| Pathway Core | *RPS6KB1* | 6 |  | 0.4281 | 0.3295 |
| Pathway Core | *RPS6KB2* | 2 |  | 0.3402 | 0.6929 |
| Pathway Core | *STK11* | 3 |  | 0.1819 | 0.0734 |
| Pathway Core | *TSC1* | 14 |  | 0.6908 | 0.5761 |
| Pathway Core | *TSC2* | 8 |  | 0.1020 | 0.0635 |
| Selenoprotein | *C11orf31* | 1 | 62 | 0.4416 | 0.1103 |
| Selenoprotein | *SELS* | 2 |  | 0.8482 | 0.2262 |
| Selenoprotein | *SEP15* | 3 |  | 0.0688 | 0.8888 |
| Selenoprotein | *SEPN1* | 5 |  | 0.7272 | 0.3877 |
| Selenoprotein | *SEPP1* | 10 |  | 0.1613 | 0.8070 |
| Selenoprotein | *SEPW1* | 3 |  | 0.4776 | 0.8243 |
| Selenoprotein | *SEPX1* | 3 |  | 0.0217 | 0.0311 |
| Selenoprotein | *TXNRD1* | 8 |  | 0.2906 | 0.9178 |
| Selenoprotein | *TXNRD2* | 21 |  | 0.7596 | 0.4666 |
| Selenoprotein | *TXNRD3* | 6 |  | 0.1579 | 0.8355 |
| Telomere | *TERT* | 7 | 7 | 0.5166 | 0.9729 |
| TGFβ | *BMP1* | 11 | 207 | 0.8735 | 0.0996 |
| TGFβ | *BMP2* | 5 |  | 0.0833 | 0.8456 |
| TGFβ | *BMP4* | 3 |  | 0.2070 | 0.6881 |
| TGFβ | *BMPR1A* | 9 |  | 0.0532 | 0.0852 |
| TGFβ | *BMPR1B* | 21 |  | 0.0693 | 0.1060 |
| TGFβ | *BMPR2* | 11 |  | 0.7706 | 0.8951 |
| TGFβ | *EIF4E* | 3 |  | 0.2264 | 0.5353 |
| TGFβ | *EIF4EBP2* | 2 |  | 0.4889 | 0.6919 |
| TGFβ | *EIF4EBP3* | 2 |  | 0.0986 | 0.2871 |
| TGFβ | *GDF10* | 7 |  | 0.9711 | 0.4621 |
| TGFβ | *RUNX1* | 40 |  | 0.1322 | 0.8356 |
| TGFβ | *RUNX2* | 19 |  | 0.7274 | 0.9898 |
| TGFβ | *RUNX3* | 9 |  | 0.1899 | 0.0244 |
| TGFβ | *SMAD1* | 5 |  | 0.9417 | 0.2159 |
| TGFβ | *SMAD2* | 4 |  | 0.3507 | 0.3957 |
| TGFβ | *SMAD3* | 37 |  | 0.0059 | 0.3554 |
| TGFβ | *SMAD4* | 2 |  | 0.7144 | 0.5281 |
| TGFβ | *SMAD7* | 11 |  | 0.4758 | 0.3207 |
| TGFβ | *TGFB1* | 2 |  | 0.2076 | 0.4922 |
| TGFβ | *TGFBR1* | 4 |  | 0.1415 | 0.1984 |
| TLR | *TLR2* | 6 | 18 | 0.0302 | 0.8110 |
| TLR | *TLR3* | 4 |  | 0.7213 | 0.7749 |
| TLR | *TLR4* | 8 |  | 0.2129 | 0.9769 |
| TNF | *NFAM1* | 12 | 31 | 0.3818 | 0.2826 |
| TNF | *NFAT5* | 8 |  | 0.3501 | 0.1336 |
| TNF | *TNF* | 2 |  | 0.9526 | 0.1166 |
| TNF | *TNFRSF1A* | 5 |  | 0.5559 | 0.8860 |
| TNF | *TRAF2* | 4 |  | 0.6786 | 0.1315 |
