# Supplementary material for: The Influence of the CHIEF Pathway on Colorectal Cancer-Specific Mortality
Source: PLoS One. 2014 Dec 26;9(12):e116169. doi: 10.1371/journal.pone.0116169 (PMC4277466; doi:10.1371/journal.pone.0116169)
Supplement: S3 Table — Genes and related SNPs associated with colorectal cancer-specific mortality among patients diagnosed with colon cancer (0.05> gene PARTP≤0.10; SNP Ptrend≤0.10). (DOCX) [file pone.0116169.s003.docx]

| Supplemental Table S3. Genes and related SNPs associated with colorectal cancer-specific mortality among patients diagnosed with colon cancer (0.05> gene P_ARTP_ ≤0.10; SNP P_trend_ ≤0.10). | | | | | |
| --- | --- | --- | --- | --- | --- |
| GENE | P_ARTP_ | SNP | Genotype | HR (95%CI) | P_trend_ |
| *BMP2* | 0.0833 | rs1979855 | TC/CC vs. TT | 0.78 (0.61, 1.00) | 0.0464 |
|  |  | rs3178250 | TC/CC vs. TT | 0.74 (0.58, 0.95) | 0.0174 |
| *BMPR1A* | 0.0532 | rs7895217 | AA vs. TT | 1.57 (1.13, 2.19) | 0.0090 |
|  |  | rs2883420 | CC vs. TT | 1.40 (1.01, 1.96) | 0.0445 |
|  |  | rs10887668 | AC/CC vs. AA | 1.40 (1.07, 1.82) | 0.0166 |
| *BMPR1B* | 0.0693 | rs10049681 | CC vs. TT/TC | 1.56 (1.10, 2.23) | 0.0186 |
|  |  | rs4699673 | GG vs. AA | 0.60 (0.32, 1.11) | 0.0357 |
|  |  | rs12508087 | AA vs. TT | 0.80 (0.43, 1.47) | 0.1000 |
|  |  | rs9307147 | GG vs. AA | 0.71 (0.49, 1.02) | 0.0582 |
|  |  | rs4490463 | GG vs. AA | 0.74 (0.52, 1.05) | 0.0588 |
|  |  | rs2120834 | CC vs. GG/GC | 1.69 (1.22, 2.34) | 0.0028 |
| *EIF4EBP3* | 0.0986 | rs250425 | TT vs. CC | 1.52 (0.92, 2.51) | 0.0594 |
| *IGF1* | 0.0611 | IGF1 | Any No 19 vs. 19/19 | 1.32 (1.03, 1.69) | 0.0242 |
| *IKBKB* | 0.0899 | rs5029748 | AA vs. CC | 1.47 (1.01, 2.14) | 0.0798 |
|  |  | rs10958713 | TT vs. CC/CT | 1.46 (1.08, 1.96) | 0.0163 |
| *IL1B* | 0.0714 | rs1143627 | CC vs. TT | 1.37 (0.96, 1.94) | 0.0306 |
|  |  | rs1143623 | CC vs. GG | 1.32 (0.87, 2.01) | 0.0182 |
| *IRF8* | 0.0675 | rs305083 | AG/GG vs. AA | 1.31 (1.04, 1.65) | 0.0230 |
|  |  | rs305080 | TT vs. CC | 1.59 (1.11, 2.29) | 0.0208 |
|  |  | rs391525 | GG vs. AA | 1.46 (0.99, 2.16) | 0.0103 |
|  |  | rs11649318 | CC vs. GG | 1.33 (0.94, 1.88) | 0.0954 |
|  |  | rs13338943 | GT/TT vs. GG | 0.78 (0.59, 1.05) | 0.0923 |
|  |  | rs10514611 | CT/TT vs. CC | 1.27 (1.02, 1.60) | 0.0367 |
|  |  | rs1044873 | CT/TT vs. CC | 1.32 (1.04, 1.68) | 0.0231 |
| *JUNB* | 0.0703 | rs2229510 | CA/AA vs. CC | 0.66 (0.41, 1.06) | 0.0706 |
| *MMP3* | 0.0693 | rs3025066 | TC/CC vs. TT | 0.70 (0.46, 1.05) | 0.0675 |
| *RPS6KA2* | 0.0618 | rs2049956 | GC/CC vs. GG | 1.33 (1.05, 1.70) | 0.0200 |
|  |  | rs1894660 | AA vs. GG | 1.49 (0.78, 2.83) | 0.0292 |
|  |  | rs6918886 | AA vs. GG | 0.76 (0.55, 1.06) | 0.0909 |
|  |  | rs932356 | CG/GG vs. CC | 0.76 (0.59, 0.98) | 0.0304 |
|  |  | rs9459715 | TG/GG vs. TT | 0.75 (0.56, 1.00) | 0.0475 |
|  |  | rs1883361 | CT/TT vs. CC | 0.80 (0.63, 1.01) | 0.0608 |
|  |  | rs4710090 | CT/TT vs. CC | 0.70 (0.56, 0.88) | 0.0022 |
|  |  | rs661325 | GC/CC vs. GG | 0.71 (0.56, 0.89) | 0.0028 |
|  |  | rs2345067 | CC vs. TT | 0.76 (0.55, 1.05) | 0.0719 |
|  |  | rs2072638 | TC/CC vs. TT | 1.29 (1.02, 1.63) | 0.0305 |
|  |  | rs1309150 | TC/CC vs. TT | 0.73 (0.57, 0.92) | 0.0074 |
|  |  | rs7745781 | AG/GG vs. AA | 1.27 (0.99, 1.62) | 0.0606 |
| *SEP15* | 0.0688 | rs9433110 | GA/AA vs. GG | 1.45 (1.07, 1.95) | 0.0195 |
| *SOCS1* | 0.0821 | rs4780355 | TC/CC vs. TT | 1.28 (1.02, 1.62) | 0.0329 |
| *STAT3* | 0.0543 | rs1053005 | AG/GG vs. AA | 0.78 (0.61, 0.99) | 0.0374 |
|  |  | rs2293152 | CC vs. GG/GC | 1.48 (1.10, 1.99) | 0.0137 |
|  |  | rs8069645 | AG/GG vs. AA | 0.79 (0.63, 0.99) | 0.0393 |
| *STAT5A* | 0.0914 | rs12601982 | GG vs. AA | 0.72 (0.37, 1.42) | 0.0410 |
| Adjusted for age, study center, race/ethnicity, sex, AJCC stage, and tumor molecular phenotype: CIMP, *KRAS*, *TP53*, and MSI. ARTP p values based on 10,000 permutations. | | | | | |
