# Supplementary material for: The Influence of the CHIEF Pathway on Colorectal Cancer-Specific Mortality
Source: PLoS One. 2014 Dec 26;9(12):e116169. doi: 10.1371/journal.pone.0116169 (PMC4277466; doi:10.1371/journal.pone.0116169)
Supplement: S4 Table — Genes and related SNPs associated with colorectal cancer-specific mortality among patients diagnosed with rectal cancer (0.05> gene PARTP≤0.10; SNP Ptrend≤0.10). (DOCX) [file pone.0116169.s004.docx]

| Supplemental Table S4. Genes and related SNPs associated with colorectal cancer-specific mortality among patients diagnosed with rectal cancer (0.05> gene P_ARTP_ ≤0.10; SNP P_trend_ ≤0.10) | | | | | |
| --- | --- | --- | --- | --- | --- |
| Gene | P_ARTP_ | SNP | Genotype | HR (95%CI) | P_trend_ |
| *BMP1* | 0.0996 | rs12114940 | GG vs. TT | 1.84 (1.16, 2.91) | 0.0141 |
|  |  | rs3924229 | TC/CC vs. TT | 1.49 (1.04, 2.13) | 0.0348 |
|  |  | rs3857979 | CC vs. TT | 1.63 (1.03, 2.58) | 0.0341 |
| *BMPR1A* | 0.0852 | rs7088641 | TC/CC vs. TT | 0.75 (0.55, 1.02) | 0.0663 |
|  |  | rs2168730 | AG/GG vs. AA | 0.67 (0.49, 0.92) | 0.0134 |
|  |  | rs7895217 | AA vs. TT | 0.63 (0.40, 1.00) | 0.0434 |
|  |  | rs4934275 | TC/CC vs. TT | 0.75 (0.53, 1.06) | 0.0945 |
| *ESR2* | 0.0857 | ESR2_Rsa | rR/RR vs. rr | 0.50 (0.25, 1.01) | 0.0342 |
| *IL1A* | 0.0537 | rs3783546 | CC vs. GG | 2.04 (1.26, 3.31) | 0.0227 |
|  |  | rs3783521 | TT vs. CC | 2.13 (1.32, 3.44) | 0.0131 |
| *IL3* | 0.0965 | rs181781 | GA/AA vs. GG | 1.42 (1.00, 2.03) | 0.0587 |
| *PRKAG2* | 0.0817 | rs1541538 | AA vs. GG | 1.63 (1.06, 2.51) | 0.0428 |
|  |  | rs2536082 | CT/TT vs. CC | 1.38 (0.96, 1.98) | 0.0881 |
|  |  | rs6947064 | GG vs. AA | 0.63 (0.37, 1.07) | 0.0709 |
|  |  | rs7805747 | GA/AA vs. GG | 0.70 (0.51, 0.97) | 0.0300 |
|  |  | rs1860743 | GA/AA vs. GG | 1.86 (1.31, 2.65) | 0.0010 |
|  |  | rs10278273 | TC/CC vs. TT | 1.40 (0.99, 1.98) | 0.0669 |
|  |  | rs7801616 | TT vs. CC | 0.61 (0.38, 0.99) | 0.0424 |
|  |  | rs7784818 | AG/GG vs. AA | 1.77 (1.21, 2.60) | 0.0020 |
|  |  | rs3934597 | CT/TT vs. CC | 0.74 (0.54, 1.01) | 0.0536 |
| *SOCS1* | 0.0965 | rs193779 | GA/AA vs. GG | 0.71 (0.52, 0.98) | 0.0378 |
| *STK11* | 0.0734 | rs8111699 | GG vs. CC | 1.44 (0.92, 2.25) | 0.0991 |
|  |  | rs7259033 | GG vs. CC | 1.46 (0.95, 2.25) | 0.0739 |
|  |  | rs741765 | AA vs. GG/GA | 1.68 (0.99, 2.83) | 0.0678 |
| *TSC2* | 0.0635 | rs2074968 | GC/CC vs. GG | 0.64 (0.47, 0.87) | 0.0052 |
| Adjusted for age, study center, race/ethnicity, sex, AJCC stage, and tumor molecular phenotype: CIMP, *KRAS*, and *TP53*. ARTP p values based on 10,000 permutations. | | | | |  |
